# Superior and inferior vena cava syndrome caused by a rare lung cancer: A case report

**DOI:** 10.1002/ccr3.9391

**Published:** 2024-08-30

**Authors:** Amey Joshi, Jason Law, Niket Shah, Harith Ghnaima, Maxwell Akanbi, Richa Tikaria

**Affiliations:** ^1^ Department of Internal Medicine, Sparrow Hospital Michigan State University Lansing Michigan USA; ^2^ Department of Oncology Mclaren Greater Hospital‐Lansing Lansing Michigan USA

**Keywords:** inferior vena cava syndrome, lung cancer, neuroendocrine tumors, radiotherapy, superior vena cava syndrome

## Abstract

Superior vena cava syndrome (SVCS) is commonly caused by mediastinal malignancies. Early identification through clinical signs and imaging is critical to avoid complications including cerebral and laryngeal edema, and cardiogenic shock. We present a case of large cell neuroendocrine carcinoma causing superior and inferior vena cava compression that responded well to radiotherapy and chemotherapy.

## 1 INTRODUCTION

Large cell neuroendocrine carcinoma (LCNEC) of the lung is a rare and aggressive subtype of neuroendocrine tumor, accounting for approximately 1%–3% of all lung cancer cases.^1^, ^2^ This malignancy is characterized by both neuroendocrine and non‐small cell lung cancer (NSCLC) features, complicating its diagnosis and treatment.

LCNEC is often diagnosed at an advanced stage, with more than half of the patients presenting with stage IV disease at the time of diagnosis. The median survival time for patients with LCNEC averages around 9.7 months from the time of diagnosis.^1^ The tumor exhibits a rapid growth pattern and a high propensity for early metastasis, contributing to its poor prognosis.

Herein, we report a rare case of LCNEC of lung primary causing superior and inferior vena cava syndrome. This case highlights the limited evidence on treating these subtypes of cancers and the challenges associated with their management.

## 2 CASE HISTORY

**FIGURE 1:**
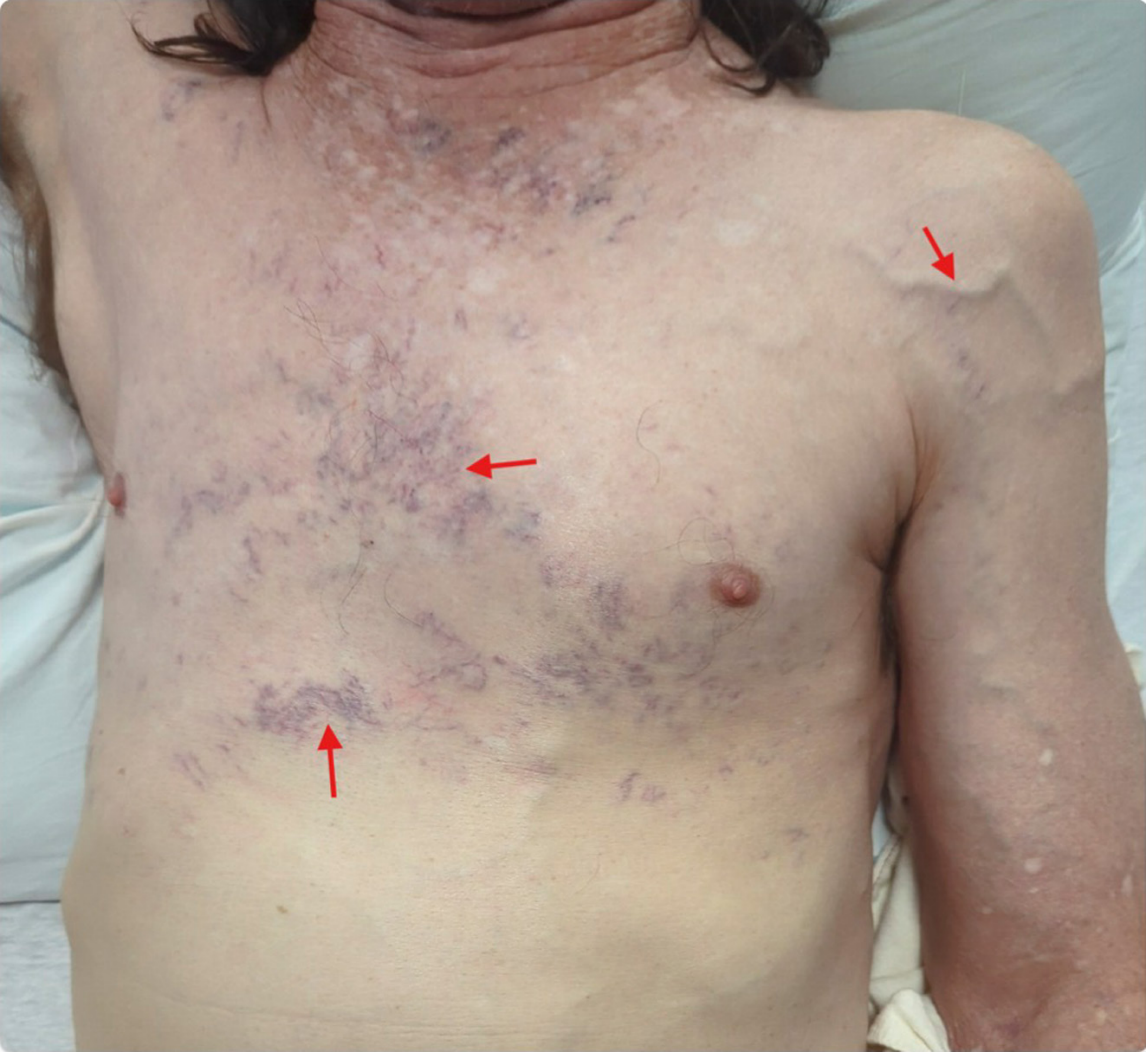
Telangiectasia (red arrows) of the upper chest and upper abdomen secondary to SVC and IVC compression.

A 50‐year‐old male with a past medical history of opioid use disorder, 30‐pack‐year history of cigarette smoking, and a significant occupational history of steel cutting presented to the emergency department with new‐onset hemoptysis and exertional shortness of breath. He reported that his shortness of breath had progressed over the preceding 4 months to be persistent at rest. The patient also endorsed significant unintentional weight loss, non‐productive cough, hoarseness of voice, and hemoptysis that was non‐reproducible. Examination revealed multiple dilated venules over the trunk above the umbilicus, distended arm and neck veins, non‐tender cervical lymphadenopathy, and non‐tender hepatomegaly (Figure 1).

## 3 METHODS

**FIGURE 2:**
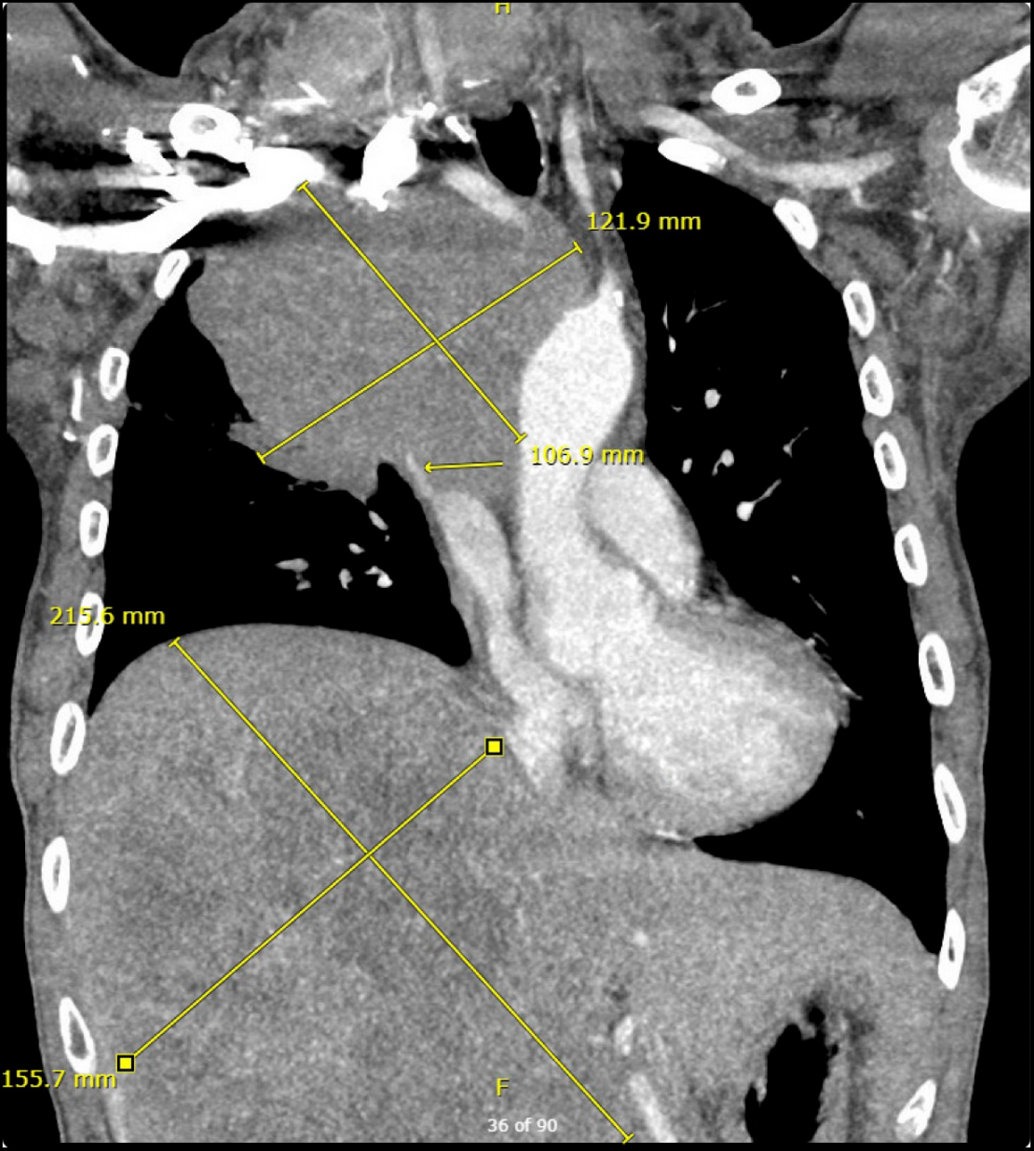
CT Chest/Abdomen/Pelvis with contrast showing a 12 × 10 cm mass occupying the right upper lobe compressing the SVC and a second mass in the liver measuring 21.5 × 15.5 cm compressing the IVC.

Laboratory investigations were significant for elevated alkaline phosphatase (687 IU/L) and gamma‐glutamyl transferase (704 IU/L). Other pertinent laboratory findings were significant for mild hyponatremia (132 meq/L), elevated d‐dimer (3 mg), elevated LDH (731 U/L). Ultrasound of the abdomen did not reveal any gall bladder or bile duct abnormalities. Hepatitis A and C antibodies and hepatitis B surface antigen was negative. Urine drug screen for hepatoxic substances, and acetaminophen levels and salicylate levels, was negative. CT with contrast of the chest and abdomen revealed a large lobulated mediastinal mass measuring approximately 12.2 × 11 cm encasing the superior and inferior vena cava, and a large solitary hepatic mass in the center of the right hepatic lobe measuring 22 × 14 × 12.6 cm (Figure 2).

A liver biopsy revealed a large cell neuroendocrine tumor. Immunohistochemical staining was positive for CK 7 (cytoplasmic and focal), synaptophysin, chromogranin, and TTF‐1, suggesting a primary lung lesion. Molecular testing revealed a high tumor mutational burden of 42%, but not targetable mutations were detected. He received radiation therapy of 400 cGy/2000 cGy over 5 days to the mediastinum before being discharged for outpatient follow‐up. The patient was treated for 6 cycles of palliative atezolizumab, carboplatin, and etoposide every 21 days as his tumor was thought to be a type II large cell neuroendocrine carcinoma. For the first 6 months Carboplatin was dosed at AUC 5 (20% dose reduced, nation shortage), etoposide at 100 mg/m^2^ days 1–3 of cycle and IV atezolizumab at 1200 mg every 21 days for the first 4 cycles. After cycle 6 maintenance Atezolizumab at 1200 mg every 21 days was initiated.

## 4 RESULTS

**FIGURE 3:**
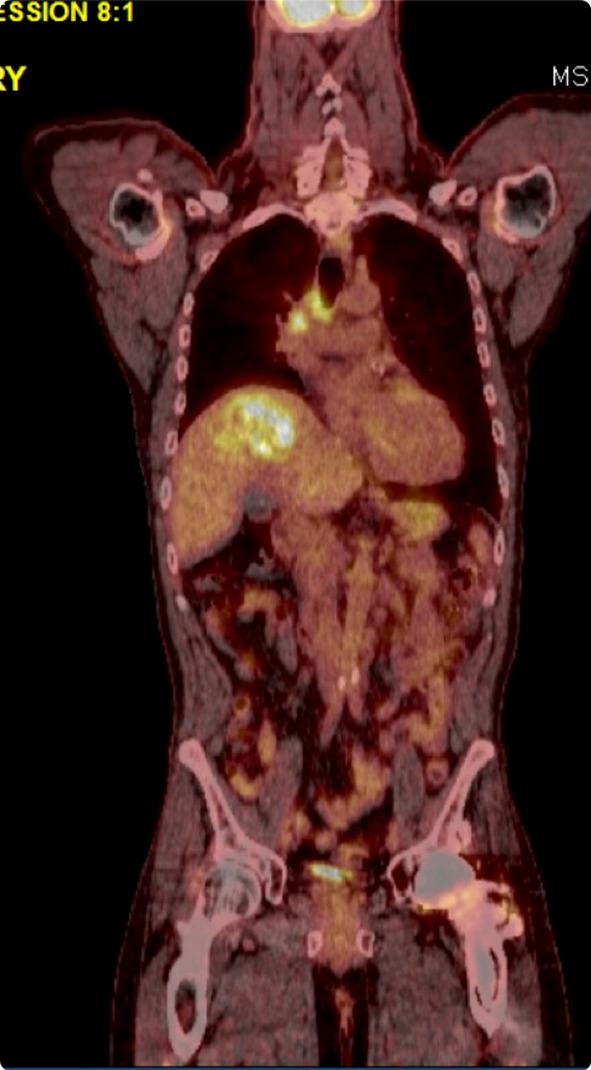
PET scan showing significant reduction in tumor size post chemoradiation.

The patient initially had some mild progression with the discovery of a new pulmonary nodule but otherwise had stable disease by Response Evaluation Criteria in Solid Tumors (RECIST) criteria at the following PET/CT (Figure 3). Symptoms of SVCS, including dilated chest veins and arm and neck swelling, were noted to be significantly reduced on follow‐up. The patient transitioned to a maintenance regimen following the completion of the palliative regimen that included atezolizumab. The patient was noted to be doing well symptomatically at an 8‐month follow‐up.

## 5 CONCLUSION

SVC and IVC invasion by lung cancer is a rare occurrence, however, can be life threatening if not timely intervened. Chemotherapy and radiotherapy can be effective in alleviating symptoms and quality of life in SVC syndrome. Endovascular stenting may play a role in certain emergent situations of SVCS. A multidisciplinary approach involving medical and surgical oncologists and intervention radiologists is required to ensure optimal treatment strategies for managing this complex medical condition.

## 6 DISCUSSION

Superior vena cava syndrome (SVCS) is a clinical manifestation arising from the partial or complete obstruction of the superior vena cava (SVC) that can be caused by a thrombus formation or tumor infiltration of the vessel wall.^1^ A majority of the SVCS are a result of mediastinal malignancies, with lung cancer being the most common. In terms of incidence, non‐small cell lung carcinomas (NSLC) are the leading cause of SVCS due to their higher prevalence. However, small‐cell lung cancers are more commonly associated with SVCS due to their central location and rapid growth. Neuroendocrine tumors are extremely rare causes of SVCS, and only a few cases have been reported worldwide.^2^

SVCS‐related and consequent collateral circulation can broadly be classified as (1) just at, (2) just above, and (3) just below the outlet of the azygos vein, depending on the cutaneous manifestations observed in the form of dilated superficial veins.^3^ In the current case, we observed dilated internal mammary, epigastric, and intercostal veins. Other features we observed in the current case were the presence of neck swelling, orthopnea, dyspnea, cough, and facial plethora, which are other common features of SVCS.^4^ Recognition of these clinical signs must prompt urgent imaging through contrast‐enhanced spiral or multi‐slice CT imaging and MRI.^1^ A biopsy of the most accessible lesion, which in the current case was the liver mass, is crucial to differentiate the type of malignancy, if any, to aid in appropriate and timely intervention.

Stenting can be considered in emergencies of impending cardiorespiratory collapse using the Kishi score (scores ≥4 are indicative of stenting therapy). The patient's Kishi score of 4 raised the question of whether the high‐risk patient would benefit from stenting versus palliative radiotherapy. After multidisciplinary consideration by medical and radiation oncology, it was determined that the patient would likely benefit from radiotherapy alone, of which he received 5 days with symptomatic improvement. Steroids have limited to no role in SVCS caused by neuroendocrine tumors and were not utilized in this case.

The treatment of neuroendocrine tumors, especially of mediastinal origin, has extremely limited evidence as to the choice of chemotherapeutic drugs and duration of radiation therapy due to the rarity of the condition.^5^ However, recent advances in molecular pathology recommend treatment schemas based on large‐cell neuroendocrine tumors (LCNET) type.^6^ Type I LCNETs are treated as non‐small cell lung cancer, and type II LCNETs are treated similarly to small cell lung cancer. The atezolizumab, carboplatin, etopiside regimen used in this case is most well studies in the SCLC population of IMPOWER‐133. The most common grade 3 and 4 adverse events in this case were neutropenia, anemia, decreased neutrophil count and thrombocytopenia. A multidisciplinary approach involving interventional radiologists and oncologists was crucial for early diagnosis and symptomatic management of SVCS in this case and in determining management. Despite newer advances in imaging techniques and chemotherapeutic drugs, survival rates for large cell neuroendocrine tumors in the lung are very poor, with a median survival rate of only 8–12 months, and further research is needed to optimize the management of the tumor and acute complications.

## AUTHOR CONTRIBUTIONS

**Richa Tikaria:** Conceptualization; investigation; project administration; supervision; validation; writing – original draft. **Maxwell Akanbi:** Conceptualization; formal analysis; investigation; methodology; resources; supervision; validation. **Jason Law:** Data curation; investigation; project administration; supervision; visualization; writing – original draft. **Amey Joshi:** Conceptualization; data curation; formal analysis; investigation; methodology; project administration; supervision; validation; writing – original draft. **Harith Ghnaima:** Conceptualization; funding acquisition; project administration; supervision; validation; visualization; writing – original draft. **Niket Shah:** Investigation; project administration; supervision; validation; writing – original draft.

## FUNDING INFORMATION

This project was funded by the Michigan State University Foundation.

## CONFLICT OF INTEREST STATEMENT

No conflict of interest.

## CONSENT

Written informed consent was obtained from the patient to publish this report in accordance with the journal's patient consent policy.

## Data Availability

The data supporting the findings of the present study are available from corresponding author upon request.
